# Morphologic evaluation of root resorption after miniscrew assisted en mass retraction in adult bialveolar protrusion patients

**DOI:** 10.1186/s13005-020-00229-z

**Published:** 2020-07-27

**Authors:** Yu Chen, Dongxu Liu

**Affiliations:** 1 Department of Stomatology, School of Medicine, Xiamen University, Xiamen, China; 2 Department of Orthodontics, School and Hospital of Stomatology, Shandong University & Shandong Provincial Key Laboratory of Oral Tissue Regeneration & Shandong Engineering Laboratory for Dental Materials and Oral Tissue Regeneration, No 44-1, Wenhua Xi Rd, Jinan City, 250012 Shandong Province China

**Keywords:** Orthodontically induced root resorption (OIRR), En mass retraction, Bimaxillary protrusion, Cone beam computer tomography (CBCT), 3D registration

## Abstract

**Background:**

Bialveolar protrusion is one of the most common chief complaints from the Asian orthodontic patients. Typical orthodontic treatment includes extraction of the bimaxillary premolars and en mass retraction of anterior tooth with maximum anchorage by placing miniscrews. However, excessive pursuit of profile improvement by retraction and intrusion of anterior teeth may result in root resorption, alveolar bone loss, even dehiscence. Thus this retrospective, analytical study was to evaluate the root resorption of anterior teeth after miniscrew assisted en mass retraction in adult bialveolar protrusion patients.

**Materials and methods:**

Thirty six adult patients with bimaxillary protrusion had four first premolars extracted, and then miniscrews were placed to provide anchorage. CBCT scans were performed before (T1) and posttreatment (T2). A new improvement project introduced for 3D CBCT registration assessment of root morphology. The paired t-test was used to compare changes from T1 to T2. The relationship between the root resorption and the movement of anterior teeth were assessed by Pearson correlation coefficient analysis.

**Results:**

The significant differences were only found in apical third of root and the largest resorption in apical third of the root is always noted in the palatal and distal sectors. Significant correlations were observed in the loss of root in distal and palatal sectors, the root length and volume decrease with the amount of anterior teeth retraction and intrusion.

**Conclusion:**

The new 3D registration assessment of root morphology will be helpful for the clinicians. Pursuit of large retraction and intrusion leads to obvious anterior teeth root resorption.

## Introduction

Bialveolar protrusion is one of the most common chief complaints in Asian orthodontic patients. Typical orthodontic treatment of bialveolar protrusion includes extraction of the 4 premolars and en mass retraction of the anterior tooth with maximum anchorage by placing miniscrews which enable maximum retraction without undesirable movements of the posterior teeth to improve the profile of patients. Meanwhile, due to the vertical component force, the anterior teeth can be intruded. However, excessive pursuit of profile improvement by retraction and intrusion of anterior teeth may result in root resorption, alveolar bone loss, even dehiscence. Our previous study already evaluates the alveolar bone loss after en mass retraction in adult bialveolar protrusion patients [1], but the retrospective orthodontically induced root resorption analysis of anterior teeth remains to be established after en mass retraction.

Orthodontically induced root resorption (OIRR) is a sterile inflammatory process and an inevitable pathological consequence of orthodontic tooth movement, and its prevalence is up to 100% in histologically examined teeth and much lower in teeth examined by routine two-dimensional radiographs [2, 3]. The extent of this inflammatory process depends on many factors. Many studies have demonstrated that biologic and mechanical factors are both important to root resorption. Biologic factors are responsible for at least 50% of root resorption and related to each patient which cannot be controlled by the clinician [4], while mechanical factors: type of appliance, displacement and type of tooth movement, magnitude and duration of force, and duration of treatment can be controlled [5–8]. The magnitude of the orthodontic force is believed to be an important factor in the etiology of OIRR, while a few studies consider the duration of force to be more important [9]. Furthermore, anterior teeth are considered to be more susceptible to OIRR than other teeth [10–12].

Previous quantitative analysis of root resorption was accomplished by using radiographs, light microscopy [3], scanning electron microscopy (SEM) [13], and micro CT [14]. OIRR is characterized by root shortening or shrinking. The 2-dimensional (2D) studies are limited to only measuring the loss of root apex, which cannot observe the loss on the root surface. In addition, magnification errors might lead to misestimation of the amounts of root resorption [15, 16]. The light microscopy, SEM, and microCT cannot be used in viviperception, which only be employed in animal experiments or assess resorption of the extracted premolars required for orthodontic purposes. However, cone-beam computed tomography (CBCT) can fill the gap, and make accurate measurements in viviperception [17–19]. For this reason, this retrospective, analytical study was to evaluate the extent of maxillary anterior teeth root resorption after en mass retraction in adult bialveolar protrusion malocclusion by CBCT 3D registration, which helps orthodontists to continue or modify the treatment plan [20].

## Materials and methods

### Patient selection

**Fig. 1:**
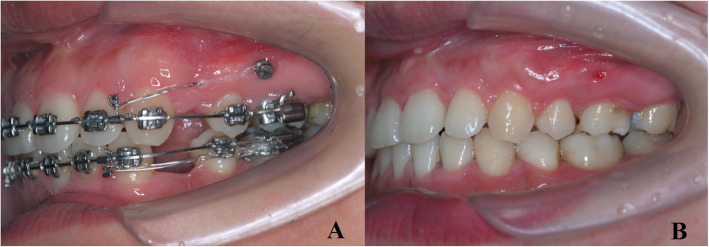
Miniscrews were placed to provide maximum anchorage: pretreatment (**a**) and post treatment (**b**)

Our study is a continuation of a series of investigations on the 3D CT registration evaluation which focuses on oromaxillo-facial function and health in adult bialveolar protrusion patients at the Shandong University in China [21–25] And the ethical issues of the research protocol were approved by Research Ethic Committee of Shandong University Dental School (No.201910005). Thirty-six bialveolar dentoalveolar protrusion patients with mild crowding were treated by extraction of the bimaxillary premolars and en mass retraction of the anterior tooth with maximum anchorage by placing miniscrews (Beici Medical Company, Ningbo, China). All patients provided informed consent and were notified of potential risks, including the damage potentially associated with CBCT radiation and miniscrew methodologies. Oriental pre-adjusted appliance KOSAKA slot brackets (OPA-K, Tomy; Fukushima-ken, Japan) were used in this study, and miniscrews were placed as an anchorage for en mass retraction. An intermittent force of 100 g per side was applied to the 4 mm crimpable hook on the distal lateral incisor with an elastic power chain extending from miniscrew. And add 700gmm moment by reverse curve spee on 0.019″*0.025″ stainless steel wire to achieve controlled tipping movement of anterior teeth (Fig. 1). The patients were seen at 1-month intervals over 20 ± 3 months to complete the treatment.

### CT scan setup and 3D models reconstruction

The skull CBCT scans were performed after implanting the miniscrews (T1) and post-treatment (T2) (KaVo Dental GmbH, Bismarckring, Germany; scan time: 8.9 s; slice thickness: 0.4 mm,120 kV, 5 mA). Then the CBCT data was saved as DICOM (Digital Imaging and Communications in Medicine) format.

**Fig. 2:**
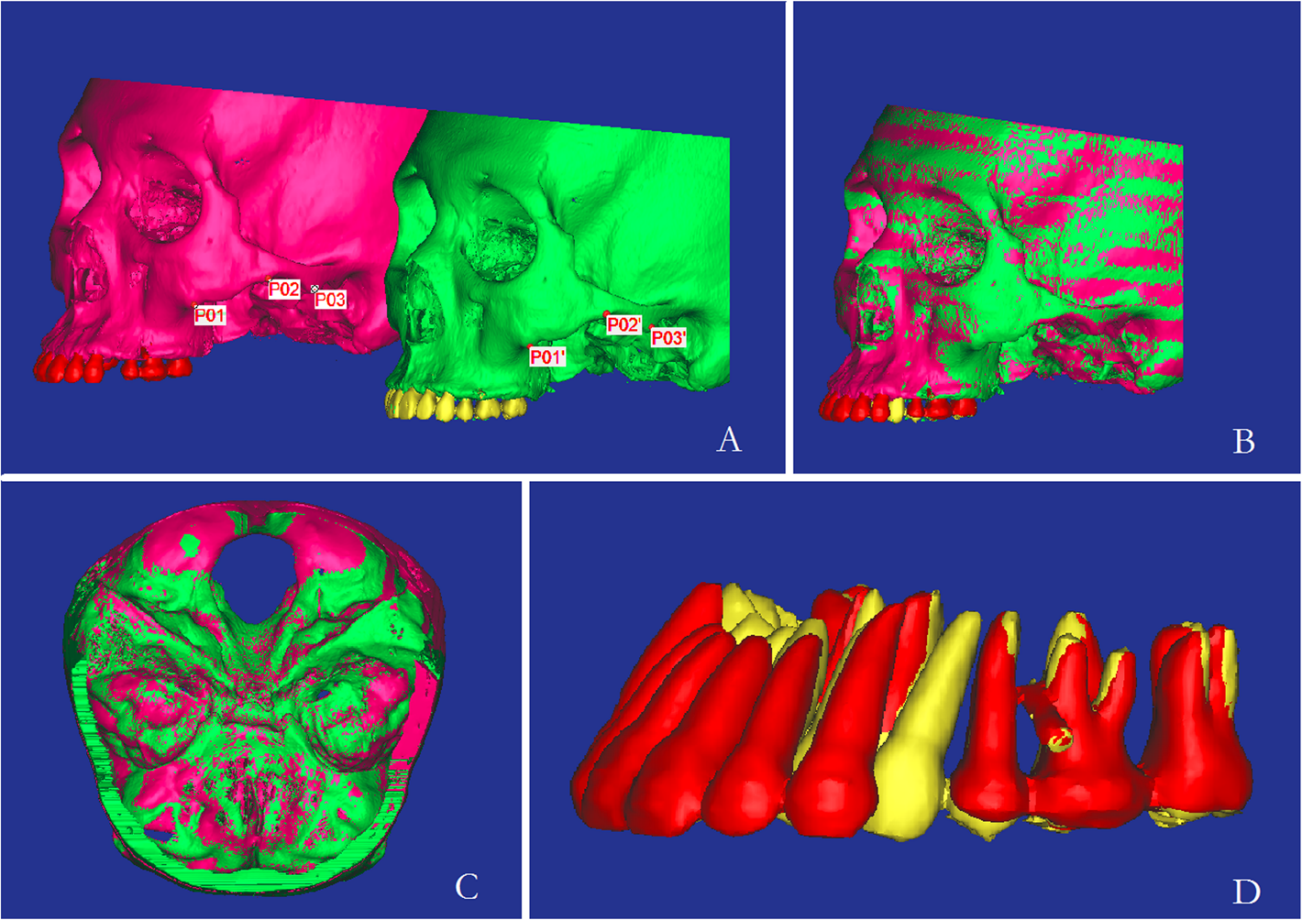
The process of point-registration and STL registration. **a** the landmarks on the zygomatic arch **b**. point-registration of pre- and post-treatment models; **c**. STL registration with cranial base; **d**. model occlusal view after registration

All three-dimensional (3D) models were constructed from CBCT data, and the bone and teeth were separated respectively according to Hounsfield Units (HU) in Materialism’s interactive medical image control system (MIMICS). Bone: 392HU ~ 1900HU; and tooth: 1500HU ~ 3725HU. The separated and independent masks were created for bone and each anterior tooth, which allowed the next generation of individual geometrical files and 3D models (Fig. 2). All 3D masks were exported as Stereo Lithography (STL) for further registration.

### Registration of pre- and post-treatment models

In MIMICS, registration was processed by a semi-automatic surface matching technique using the landmarks on the models. Laying landmarks on the zygomatic arch inferior margin of pre- and post-treatment models for Initial registration, STL was moved to a certain location by point-registration. Then, STL-registration was performed to place STL on CT-mask to improve accuracy (Fig. 2). In order to ensure the precision, corresponding landmarks were identified repeatedly (minimal point distance filter was 0.10 mm, which was satisfied as Fig. 2c). Similarly, the registration of teeth is also completed by these 2 steps above. And the landmarks of the incisor teeth are right and left incisal angle and cingulum. The landmarks of canine are right and left adjacent points and cusp. All the registration was done three times in two weeks and the best one was chosen for measurements [22–25].

### 3D measurement

**Fig. 3:**
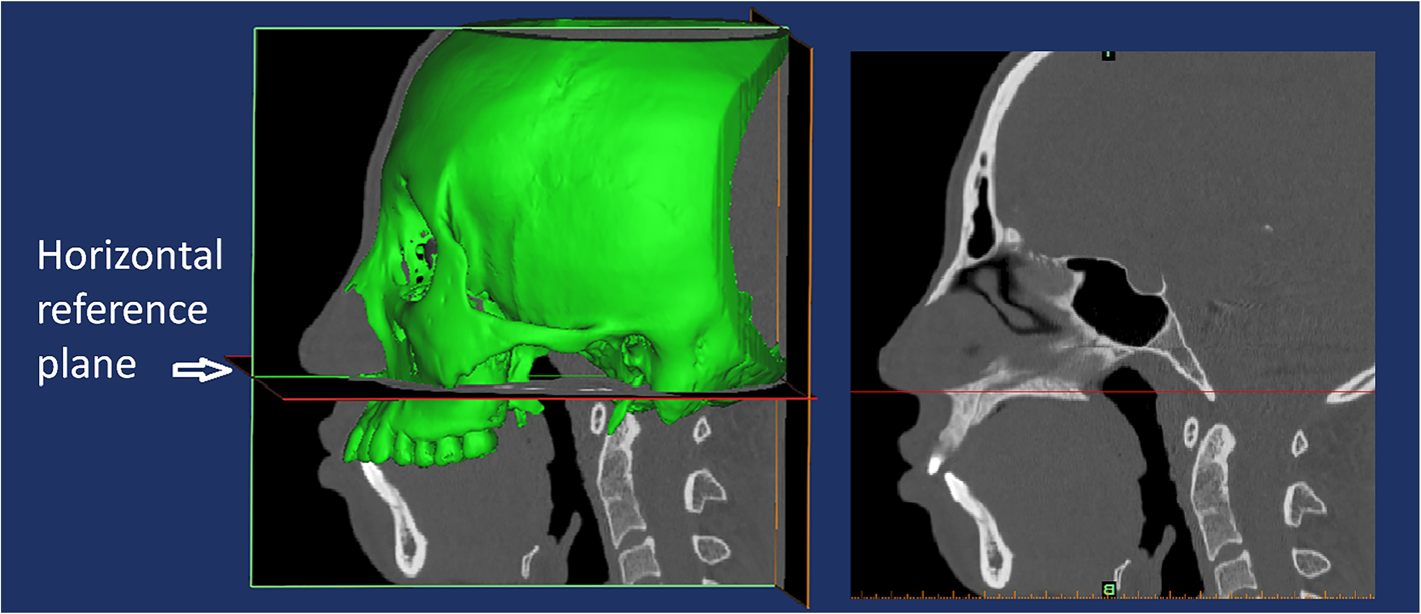
horizontal reference plane was palatal plane

**Fig. 4:**
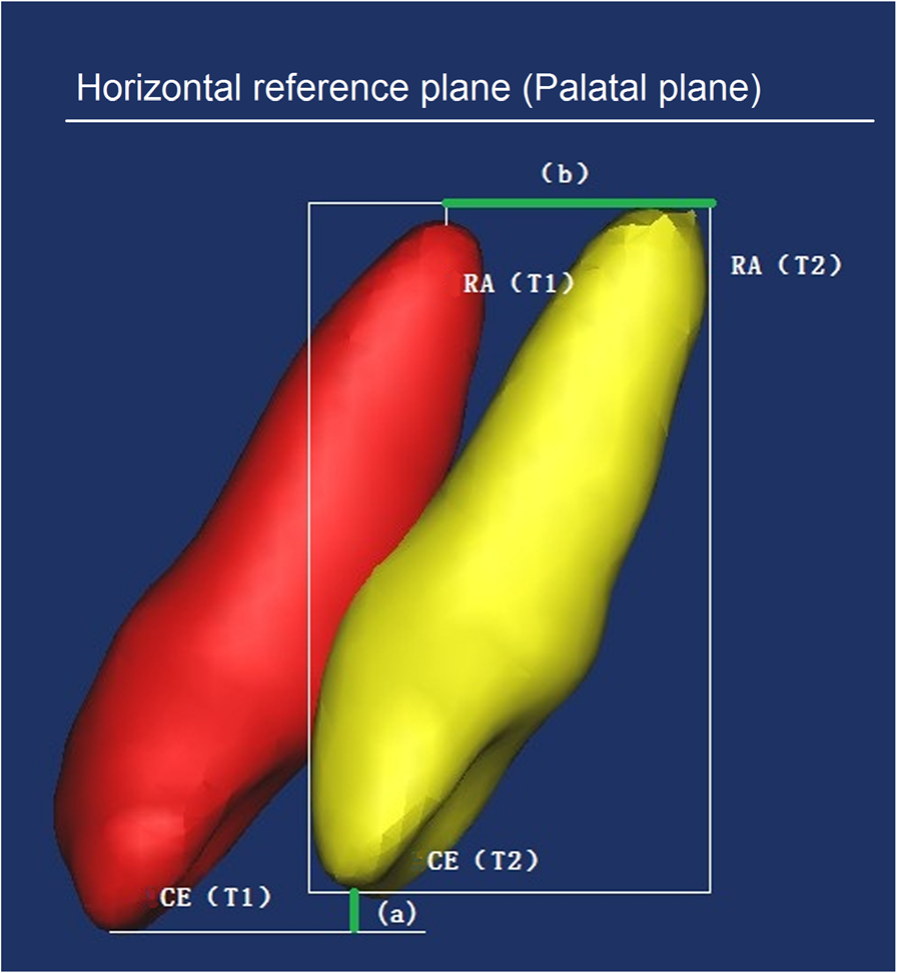
Movement of teeth between T1 and T2 models were measured

**Fig. 5:**
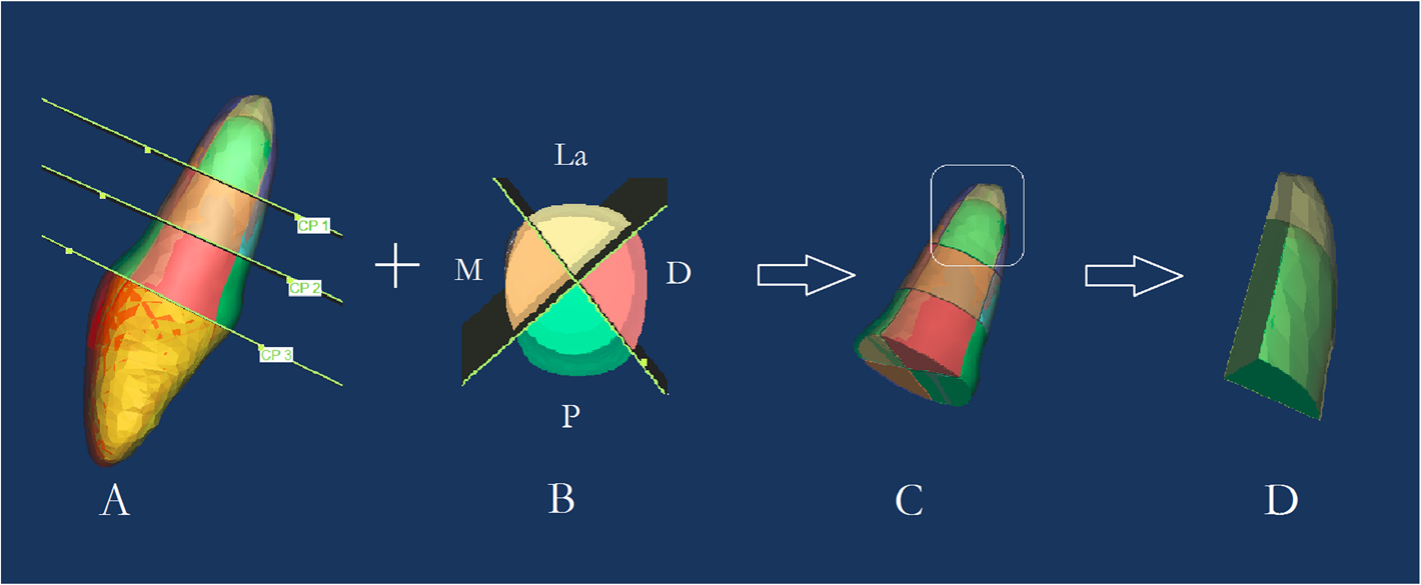
The segmentation of each tooth. **a** The root was divided into vertical thirds: cervical, middle and apical thirds **b** two perpendicular planes along mesial-distal and labial-palatal direction divide each tooth into four sectors **c** tooth is divided 12 segments **d** enlarged drawing of C

In this study, the root was divided into vertical thirds by two parallel planes which were perpendicular to the axis of tooth: cervical, middle and apical thirds (Fig. 5a). Along the axis of the tooth, we set two perpendicular planes along mesial-distal and labial-palatal direction, and each anterior tooth was divided into four sectors: labial sector (La), palatal sector (P), mesial sector (M), distal sector (D) (Fig. 5b). We measured the changes of volume as the final results of the 12 parts of root resorption amount. In order to evaluate the movement of the anterior teeth, the horizontal reference plane was palatal (Fig. 3). The landmarks identified on each 3D model were: midpoint of crown edge (CE), root apex (RA) (Fig. 4). The variables measured on each 3D model were shown in Table 1. Due to the root resorption, the apex of the root cannot be set as a reference point to assess the vertical movement of teeth. Thus we put the reference point on the central incisor to measure the amount of retraction and intrusion of the anterior teeth. Every subject was measured three times by the same investigator and then averaged.

**Table 1:** Measurement Variables of Teeth Used

Measurement Variables	Definition
CE (T1—T2) (a)	anterior teeth retraction amount at crown edge in the vertical direction
RA (T1—T2) (b)	anterior teeth retraction amount at root apex in the horizontal direction
La (T1—T2)	Loss of root on labial surface
P (T1—T2)	Loss of root on palatal surface
M (T1—T2)	Loss of root on mesial surface
D (T1—T2)	Loss of root on distal surface
RA (T1—T2)	Loss of root at root apex

### Statistical analysis

All statistical analyses were performed by the SAS software package (version 9.13, SAS Institute, Cary, NC). For each variable measured on the 3D models, the mean and the standard deviation were calculated. Length and volume Changes of the root between T1 and T2 were assessed by using paired t-tests. Next, the relationship between the root resorption and the movement of anterior teeth were assessed by Pearson correlation coefficient analysis. The error of the method based on double measurements at a 2-month interval was performed on 12 randomly patients for 3D linear and volume measurements was calculated as s = √Σ (d) ^2^/2n (d for deviations between the 2 measurements; n for the number of paired objects) [26]. The error was 0.163 mm (SD of d is 0.042 mm) for 3D linear measurement and was 0.123 mm^3^ (SD of d is 0.066 mm^3^) for root volume measurement. The statistical difference was not significant between two measurements by paired t-test at the significance level α = 0.05.

## Results

There is no significant difference in root length and volume changes between the left and right side corresponding contralateral teeth (*P* > 0.05), so left anterior teeth were chosen to do the further statistical analysis. Using the paired t-test, only significant volume differences between T1 and T2 were found in the apical third of root (P<0.05). Thus, the apical third of root was chosen as the object of study. Then using the paired t-test, letting the significance level α = 0.05 and determining the value of t_0.05_(n-1) = t_0.05_ (35) =1.689 based on the t-distribution with n-1 degrees of freedom, there was a significant difference if t_i_>t_0.05_ (35), i = 1, 2, 3, 4 where t_i_ is the sample value of t-test statistic based on 36 sample values in mesial, distal, labial and palatal sectors. We computed t_4_>t_2_>t_1_>t_3_>t_0.05_ (35) in central incisors and t_4_>t_2_>t_3_>t_1_>t_0.05_ (35) in lateral incisors and canines, namely, we concluded that there was a significant difference in every sector of root apical third, and there were greater amounts of root resorption in the palatal and distal sectors compared with the mesial and labial sectors in the apical third.

In addition, anterior teeth were divided into 3 groups: central incisors, lateral incisors, and canines. The apical third root resorption in mesial, distal, labial palatal sectors, and the decrease of root length and volume are shown in Table 2. Although there is no significant difference in root resorption volume between lateral incisors and canines (*P* = 0.585>0.05) by independent-samples t-test, the greatest decrease of root length in the anterior teeth is always occurred in the lateral incisors (1.475 ± 0.380 mm). The amounts of anterior teeth retraction at edge and intrusion at the edge were 6.097 ± 0.973 mm and 3.353 ± 0.305 mm, respectively. For all anterior teeth, no significant correlation was observed in the loss of root in mesial and labial sectors with the amount of anterior teeth retraction and intrusion. A significant correlation was observed in the loss of root in distal and palatal sectors, the root length and volume decrease with the amount of anterior teeth retraction in all anterior teeth. And a significant correlation was found in the amount of anterior teeth intrusion with the root length decrease and the loss of root in the distal sector in central Incisors, with the root length decrease in lateral Incisors, and with the root length and volume decrease and the loss of root in the palatal sector in canines (Table 3).

**Table 2:** The apical third root resorption in mesial, distal, labial palatal sectors and the decrease of root length (mm) and volum (mm^3^)

groups		mesial	distal	labial	palatal	root length	root volum
central incisors	mean	0.949	1.711	0.828	2.661	0.974	6.150
	SD	0.246	0.509	0.151	0.968	0.204	1.364
lateral incisors	mean	1.529	2.223	1.828	4.622	1.475	10.203
	SD	0.248	0.283	0.325	0.400	0.380	0.596
canines	mean	1.360	2.846	1.486	4.423	1.084	10.114
	SD	0.235	0.4095	0.239	0.372	0.258	0.756

**Table 3:** Pearson correlation coefficient analysis between the root resorption and some parameters

variable			The amount of retraction	The amount of intrusion
Central Incisors	Mesial sector	r	−0.146	0.244
		p	0.397	0.152
	Distal sector	r	0.451*	0.633*
		p	0.006*	0.000
	Labial sector	r	0.140	0.061
		p	0.417	0.724
	Palatal sector	r	0.082	0.624
		p	0.634	0.000
	Length of root	r	0.495*	0.664*
		p	0.002	0.000
	Volume of root	r	0.216	0.730*
		p	0.206	0.000
Lateral Incisors	Mesial sector	r	−0.151	−0.192
		p	0.378	0.262
	Distal sector	r	0.221	0.708*
		p	0.195	0.000
	Labial sector	r	−0.161	−0.289
		p	0.349	0.087
	Palatal sector	r	0.140	0.594*
		p	0.415	0.000
	Length of root	r	0.518*	0.487*
		p	0.001	0.003
	Volume of root	r	0.048	0.497*
		p	0.779	0.002
Canines	Mesial sector	r	0.030	0.129
		p	0.860	0.452
	Distal sector	r	0.287	0.740*
		p	0.090	0.000
	Labial sector	r	−0.069	−0.261
		p	0.690	0.125
	Palatal sector	r	0.400*	0.597*
		p	0.016	0.000
	Length of root	r	0.692*	0.409*
		p	0.000	0.013
	Volume of root	r	0.340*	0.653*
		p	0.043	0.000

*p < 0.05

## Discussion

Many factors affect root resorption: orthodontic force level, treatment type, and method of quantification of root resorption, which was difficult to control in previous studies. In our study, we had comparable clinical subjects: similar bialveolar dentoalveolar protrusion patients with mild crowding who need strong anchorage for anterior teeth retraction, similar orthodontic load with miniscrews was demonstrated the finite element method and more reliable 3D registration assessments of root resorption by CBCT. CBCT can provide the possibility of assessing root surfaces that are not displayed on conventional radiographs. Therefore, CBCT is chosen to reconstruct the extent of root resorption of anterior teeth after en mass retraction in adult bialveolar protrusion patients before and post-treatment. And the 3D registration assessments of root resorption in our study (Fig. 5) not only can be used in orthodontically induced root resorption but also can be used for assessing other external apical root resorption and root development, such as the root morphology of impacted tooth, which is very important in treatment planning, for the impacted site and the severity of root are the determinants of the extraction of the impacted tooth. In addition, the software company can introduce a teeth standard model for 3D registration of impacted tooth, which can provide 3D visual assessments of the tooth. Furthermore, the standard model can be corrected by inputting the data of contralateral homonymous healthy teeth individually. It will be useful for primary hospital clinicians who have not received training in the interpretation of CBCT images to make their clinical decision.

The changes in cervical, middle and apical thirds between pre-and post-treatment are measured in our study, and only the resorption in apical third was observed, which is inconsistent with the findings of other studies, showing that there was a difference in the apical and cervical thirds [13]. One cause is that tipping, extrusion, and intrusion forces resulted in the greatest stress at the root apex [27]. Meanwhile, the apical third of root is more susceptible to root resorption than the other two parts. Hohmann et al. suggested that the different blood sources for vascularization of the periodontal ligament in both parts might be related [28], and Srivicharnkul P suggested that apical cementum is softer than cervical cementum, because of fewer Sharpey’s fibers, may lead to more resorption of the root apex [29]. The other cause may be the resolution of CBCT, which cannot note the adequate changes in cervical thirds. Therefore, it is worth mentioning that the real root resorption amount is as obvious as what has been shown in CBCT at least.

In addition, a significant root resorption was found at 4 sectors of the root between pre-and post-treatment, and in the descending order, they were palatal sectors, distal sectors, labial sectors, and mesial sectors. Using Pearson correlation coefficient analysis, a significant correlation was observed in the amount of anterior teeth retraction and the loss of root in distal and palatal sectors, pointing out that pressure in the periodontal ligament was greater in the palatal and distal sectors than in the labial and mesial sectors during en mass retraction by sliding mechanics. Orthodontically induced inflammatory root resorption is associated with local compression of the periodontal membrane. Overcompression of the periodontal ligament will result in tissue hyalinization. The nearby cementum and cementoid can be damaged during the removal of the hyaline zone [30].

As expected, root length and volume decreased obviously in each group. And using Pearson correlation coefficient analysis, there was a significant correlation in the number of anterior teeth intrusion and retraction with the loss of root length at the apex, showing that great pressure in the periodontal ligament was at the root apex, which is consistent with the findings of other studies, and it also implied that the loss of root length is a sensitive indicator for root resorption, even though it is a 2D measurement. Additionally, the greatest loss at the root apex was always noted in lateral incisors. One factor contributing to this is that the force of elastic chains was applied from miniscrew to the upper crimpable hook on the distal lateral incisor to retract and intrude the upper anterior tooth. Although the elasticity modulus of stainless steel is higher than 10 times of the dental tissue, it is still lower than the ideal rigid body. Torsional deflection will occur under 150 g force, and it is mainly focused on the tooth near the force point. Therefore, lateral incisors and canines suffered greater pressure than other teeth, which is in good agreement with other studies, using a 3-D finite element method to simulate en mass retraction of upper anterior teeth with miniscrew as anchorage [31]. And the other reason is that lateral incisor has the thinnest root in anterior teeth.

As we know, the distance between apical constriction and anatomical apical range from 0.5 mm to 1 mm, and it may be more than 1 mm to the old because of excementosis [32]. In our study, the root resorption at apical third is about 1 mm, which may lead to the damage of apical constriction, the important anatomic structure of the root. Therefore, to obtain appropriate response during en mass retraction in adult patients, we must pay more attention to the force magnitude and direction to accomplish our goal of maintaining the health, function, and aesthetics in the orthodontic treatment.

It is known that an intermittent controlled orthodontic force or a reduction of force below a certain level allows reparative mechanisms of root [33]. However, whether cementum will repair the root and the extent of reparation in this investigation are unknown, which needs further long-term research.

## Conclusion

We introduce a new improvement project for the 3D registration assessment of root morphology, which will be very helpful for the clinicians. And the mechanobiological response of the root should be taken into consideration during large en mass retraction, and the pursuit of large retraction and intrusion might lead to obvious orthodontically induced root resorption in bialveolar protrusion adult patients, which may compromise the benefits of a successful orthodontic outcome.

## Data Availability

All data generated or analyzed during this study are included in this published article.
